# Digital Technology in Psychiatry: Survey Study of Clinicians

**DOI:** 10.2196/33676

**Published:** 2022-11-10

**Authors:** William Andrew Sterling, Michael Sobolev, Anna Van Meter, Daniel Guinart, Michael L Birnbaum, Jose M Rubio, John M Kane

**Affiliations:** 1 Institute of Behavioral Science Department of Psychiatry Zucker Hillside Hospital Glen Oaks, NY United States; 2 The Donald and Barbara Zucker School of Medicine Hofstra University Northwell Health New York, NY United States; 3 Department of Psychiatry Grossman School of Medicine New York University Langone Health New York, NY United States; 4 Center for Psychiatric Neuroscience Feinstein Institute for Medical Research Manhasset, NY United States

**Keywords:** digital psychiatry, passive monitoring technology, digital phenotype, psychiatry, mental health, clinicians, clinician perspectives, digital health, physicians, psychiatrists

## Abstract

**Background:**

Digital technology has the potential to transform psychiatry, but its adoption has been limited. The proliferation of telepsychiatry during the COVID-19 pandemic has increased the urgency of optimizing technology for clinical practice. Understanding clinician attitudes and preferences is crucial to effective implementation and patient benefit.

**Objective:**

Our objective was to elicit clinician perspectives on emerging digital technology.

**Methods:**

Clinicians in a large psychiatry department (inpatient and outpatient) were invited to complete a web-based survey about their attitudes toward digital technology in practice, focusing on implementation, clinical benefits, and expectations about patients’ attitudes. The survey consisted of 23 questions that could be answered on either a 3-point or 5-point Likert scale. We report the frequencies and percentages of responses.

**Results:**

In total, 139 clinicians completed the survey—they represent a variety of years of experience, credentials, and diagnostic subspecialties (response rate 69.5%). Overall, 83.4% (n=116) of them stated that digital data could improve their practice, and 23.0% (n=32) of responders reported that they had viewed patients’ profiles on social media. Among anticipated benefits, clinicians rated symptom self-tracking (n=101, 72.7%) as well as clinical intervention support (n=90, 64.7%) as most promising. Among anticipated challenges, clinicians mostly expressed concerns over greater time demand (n=123, 88.5%) and whether digital data would be actionable (n=107, 77%). Furthermore, 95.0% (n=132) of clinicians expected their patients to share digital data.

**Conclusions:**

Overall, clinicians reported a positive attitude toward the use of digital data to not only improve patient outcomes but also highlight significant barriers that implementation would need to overcome. Although clinicians’ self-reported attitudes about digital technology may not necessarily translate into behavior, our results suggest that technologies that reduce clinician burden and are easily interpretable have the greatest likelihood of uptake.

## Introduction

Digital technology is a central feature of modern life and is becoming an increasingly prominent component of modern medical practice. In psychiatry, there is established evidence that digital data can be used to monitor multiple health outcomes. Metrics including the frequency of web-based activity, the content and language uploaded to social media, and smartphone sensing of biometrics including sleep and physical activity have strong predictive value for medication compliance, current mental status, or risk of relapse and can be used to guide treatment decisions effectively [1-5]. Mental health apps show broad applications across many patient demographics and diagnostic categories [6]. The integration of digital data into clinical practice represents not only a new means of interacting with patients—allowing clinicians to monitor dynamic symptoms in real time, providing patients and clinicians alike early notice of relapse and reducing latency during intervention—but also, indeed, a new way of understanding patients, thus synthesizing symptoms data into individualized profiles for each unique patient who uses the technology—a digital phenotype [7]. However, while academic research has explored patients’ digital phenotypes, this work has not yet fully integrated multiple streams of digital data, nor has it been deployed systematically in actual clinical settings [8].

Clinician attitudes toward the use of digital data in psychiatry—in terms of benefits to clinical care, barriers to effective implementation, understanding of the capabilities of current technology, and willingness to change current practice—are a key factor in the development and adoption of future technological platforms, but they remain underexplored and underappreciated [9-11]. Indeed, clinician enthusiasm has been shown to be a vital factor to successful implementation of new digital platforms in clinical settings [12]. Studies of telepsychiatry suggest that successful implementation of digital technology into clinical settings depends on clinician enthusiasm and confidence in the safety and efficacy of the new platform, as well as access to proper training [13-15]. Previous studies of clinicians’ attitudes toward digital data in psychiatry indicate some enthusiasm regarding administrative improvements that technology could provide—including ease of scheduling and monitoring patients between sessions—but strong concerns about privacy, and limited understanding of the capabilities of digital platforms has been demonstrated previously [16]. Elsewhere, surveys of clinicians have demonstrated a negative correlation between professional experience and clinicians’ attitudes toward the use digital data in psychiatry [17]. To develop a successful platform for clinicians to use digital data in practice, researchers must determine not only clinicians’ attitudes toward the technology but also their literacy about its efficacy and potential as a clinical aid.

Although digital mental health data have been researched for over a decade, their development has recently become urgent owing to the COVID-10 pandemic. Clinical practices worldwide experienced a massive shift toward remote assessment as a result of COVID-19 restrictions, and clinicians were rapidly introduced to telepsychiatry, who under, normal circumstances may never have been exposed to it. This shift in practice highlighted not only the need for increased clinical services for individuals without access but also the reluctance of many clinicians to engage with telehealth technologies owing to concerns about its efficacy and compromised privacy [18,19]. As future clinical practice in psychiatry may continue to rely on remote assessment, understanding clinician perspectives on this technology remains crucial to optimizing service and user experience.

The aim of our study was to capture clinician attitudes and expectations regarding emerging digital technology in psychiatry, in light of the unprecedented shift in clinician experience that the COVID-19 pandemic represents. Previously reluctant or unfamiliar clinicians will now have had significant experience of telehealth practice, and attitudes toward digital data in psychiatry may have shifted. Understanding clinician attitudes and preferences is necessary to overcome implementation challenges and achieve patient benefits.

## Methods

### Methods Overview

The survey was developed for the purpose of this study by the Digital Clinic research group, which is composed of clinicians working in outpatient and inpatient settings as well as dedicated research staff familiar with emerging digital technology in psychiatry, working on the implementation of digital technology in clinical settings across our health system. The survey was designed to study clinician attitudes toward the use of digital technology in psychiatry in a variety of dimensions using varied Likert scales. Questions were written on the basis of expert research group members’ existing knowledge of digital technology in psychiatry as described in current literature, and in anticipation of developing a digital data platform for use in our campus’ outpatient clinic (see attached survey in Multimedia Appendix 1).

Survey items included (1) an assessment of whether clinicians thought digital data about patients could inform their practice (using a 5-point Likert scale: 0%=”No,” 25%=“I’m not sure, I need to know more,” 50%=“I think so, but would need to try it out,” 75%=“Yes,” and 100%=“Definitely, I incorporate this data already”), (2) ratings of perceived relative usefulness of different types of digital patient data (such as sleep, physical activity, location, web-based search activity, etc, using a 3-point Likert scale: 0%=“Low,” 50%=“Medium,” and 100%=“High”), (3) anticipated barriers to using digital data in clinical practice (such as “Patient participation,” “Increased time demands tending to flagged digital events,” “Volume of data created by digital monitoring,” etc, using a 3-point Likert scale: 0%=“Not a barrier,” 50%=“Somewhat of a barrier,” and 100%=“A significant barrier”), (4) anticipated benefits to using digital data in clinical practice (such as “Having a consistent source of collateral data,” “Helping patients feel better understood,” “As an alert system when patient activities change,” etc, using a 3-point Likert scale: 0%=“Not a benefit,” 50%=“Somewhat of a benefit,” and 100%=“A significant benefit”), (5) clinicians’ expectations of whether patients would be willing to share digital data in a clinical setting and experience using digital patient data from social media (using multiple-choice questions about how data were used and free-text responses to allow responders to elaborate), (6) rating level of agreement with statements about the incorporation of digital data into psychiatric practice (such as “Having access to information collected in a Digital Clinic will... Lead to more frequent patient encounters,” “...Improve clinical outcomes,” or “...Increase the amount of documentation to complete,” using a 5-point Likert scale: 0%=“Strongly disagree,” 25%=“Disagree,” 50%=“Neither agree nor disagree,” 75%=“Agree,” and 100%=“Strongly agree”), (7) assessment of if or how clinician attitudes toward digital data in psychiatry had changed in response to the COVID-19 pandemic and the changes in practice that many of the surveyed participants had experienced (using free-text responses), and (8) demographic characteristics (including age, years in practice, nature of practice, and role in clinic).

The survey was administered to all clinicians on the campus of an academic psychiatric treatment facility. The facility surveyed here is in a demographically diverse section of a major urban area, with high access to mental health services. The survey was written in Survey Monkey and administered during a Grand Rounds event. It was subsequently emailed to all clinicians on campus. A total of 200 clinicians were surveyed—150 outpatients and 50 inpatients. The survey was open for 6 months from May to November 2020. Participation in the survey was voluntary. There was no compensation for completing the survey. Responders were given the option to share their contact information (deanonymizing themselves) to participate in a possible focus group, but owing to the COVID-19 pandemic, this focus group was not held.

### Ethical Considerations

The survey was reviewed by the Northwell institutional review board and granted an exemption (#19-0958).

### Statistical Analysis

Descriptive statistics were used to report survey results. Chi-square tests were used to compare categorical variables. First, omnibus comparisons were conducted by prescribing status (yes/no). If significant differences were detected, we then tested the individual interactions of interest post hoc. Wilcoxon tests for nonnormally distributed, continuous variables (age), as determined by a Shapiro–Wilk W test, were conducted. All analyses were conducted using JMP, (version 13; SAS Institute Inc, 1989-2019).

## Results

### Overview

We received a total of 139 completed survey responses (response rate 69.5%). The median age of responders was 42 (IQR 34-70) years, with a median 12 (IQR 4-33) years of clinical experience. In total, 50 (36.0%) responders were psychiatrists, 27 (27.0%) were residents, 20 (14.4%) were psychologists, 14 (10.1%) were social workers, 3 (2.2%) were nurse practitioners, and 25 (16.5%) were nurses or other clinical staff. Furthermore, 79 (56.8%) responders provided medication management and 82 (59.0%) provided individual psychotherapy (Table 1).

**Table 1:** Characteristics of responders (N=139).

Characteristics		Value
Age (years), median (IQR)		42 (34-70)
Years in practice, median (IQR)		12 (4-33)
**Gender, n (%)**		
	Male	53 (38.1)
	Female	74 (53.2)
	Declined to answer	12 (8.7)
**Role in clinic, n (%)**		
	Psychiatrist	50 (36.0)
	Resident or intern	27 (19.4)
	Psychologist	20 (14.4)
	Nurse practitioner	3 (2.2)
	Nurse	2 (1.4)
	Social worker	14 (10.1)
	Other	23 (16.6)
**Type of practice (allowed to select more than one), n (%)**		
	Medication Management	79 (56.8)
	Individual Psychotherapy	82 (59.0)
	Group Therapy	30 (21.6)
	Neuromodulation	5 (3.6)
	Administration	26 (18.7)
	Research	3 (2.2)

### Attitudes Toward Digital Data in Psychiatry

Out of 139 responders, 116 (83.4%) stated that digital data could improve their clinical practice. Among different categories of digital data, responders rated data on Sleep (n=100, 72.0%), treatment adherence (n=101, 79.5%), substance use (n=105, 76.0%), self-reported symptom ratings (n=90, 70.0%), and physical activity (n=73, 53%) as having the highest potential utility in practice. Responders rated location (n=33, 26.0%), screen time (n=34, 24.0%), and criminal justice data (inmate registries, WebCrims, etc; n=25, 18.0%) as having the lowest potential use in practice (Table 2).

**Figure 1:**
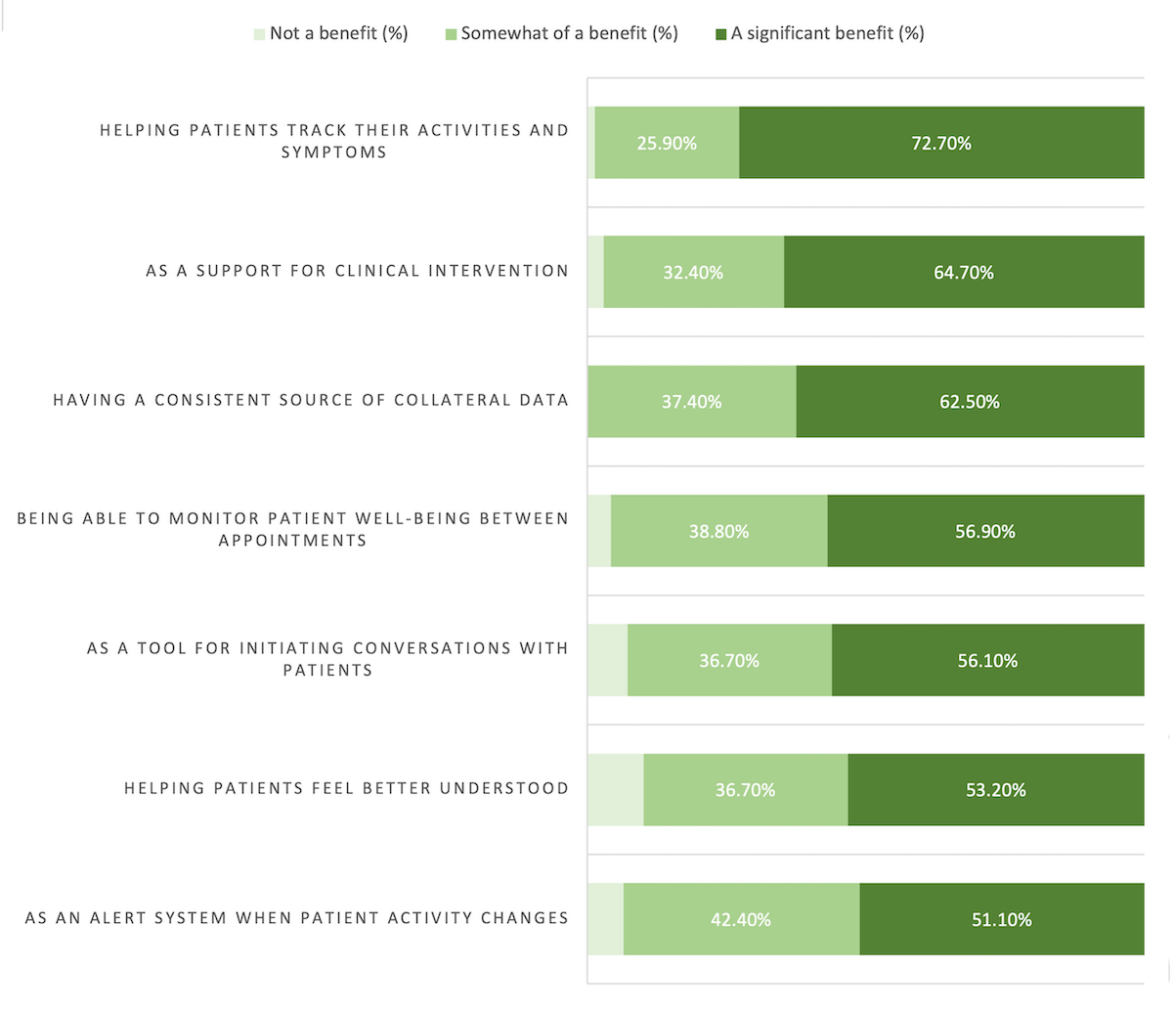
Clinicians' responses to the question, "What do you anticipate would be the greatest benefits of digital data?".

**Figure 2:**
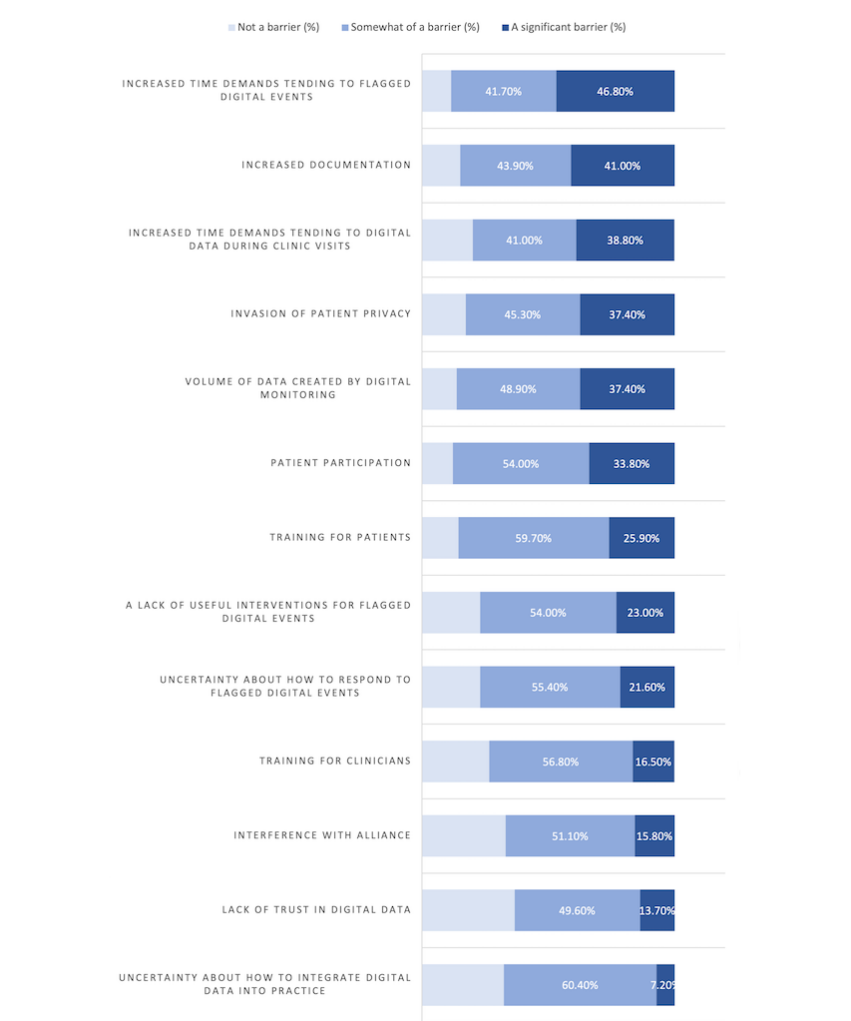
Clinicians' responses to the question, "What do you anticipate would be the greatest barriers against your use of digital data?".

Among anticipated benefits, responders rated “Helping patients track their activities and symptoms” (n=101, 72.7%), “As a support for clinical intervention” (n=90, 64.7%), and “Having a consistent source of collateral data” (n=86, 62.5%) as offering the most significant benefit. Responders rated “Helping patients feel better understood” as the least potentially beneficial aspect of using digital data in psychiatry (“Not a benefit” n=14, 10.1%; Figure 1). Among anticipated barriers to the use of digital data, responders rated “Increased time demands tending to flagged digital events” (n=54, 38.8%), “Volume of data created by digital monitoring” (n=52, 37.4%), “Increased documentation” (n=57, 41%), “Patient participation” (n=46, 33.8%), and “Increased time demands tending to digital data during clinic visits” (n=54, 38.8%) as the most likely barriers. Responders rated “Lack of trust in digital data” (n=51, 36.7%), “Interference with alliance” (n=46, 33.1%), and “Uncertainty about how to integrate digital data into practice” (n=45, 32.4%) as the least likely barriers (Figure 2). Overall, 74.1% (n=103) of responders stated that they would consult a dashboard of patient digital data prior to a clinic visit, and 85.6% (n=119) of them stated that they thought it would be beneficial for patients to have access to an app-based dashboard with information about their digital data.

**Table 2:** Attitudes toward digital data in psychiatry.

Rating of the relative level of perceived usefulness that each category has for individuals’ practice	Low, n (%)	Medium, n, (%)	High, n (%)
Sleep	3 (2.2)	27 (19.4)	100 (71.9)
Physical activity	4 (2.9)	59 (42.2)	73 (52.5)
Social media activity (frequency of posts or content of posts concerning symptoms of mental illness)	13 (9.4)	69 (49.6)	57 (41.0)
Web-based search activity (content of which concerns symptoms of mental illness)	19 (13.7)	58 (41.7)	62 (44.6)
Mobility (time spent away from home versus at home)	14 (10.1)	59 (42.4)	66 (47.5)
Location	36 (25.9)	65 (46.8)	33 (23.7)
Substance use	10 (7.2)	20 (14.4)	105 (75.5)
Screen time	34 (24.5)	66 (47.5)	38 (27.3)
Treatment adherence	7 (5.0)	22 (15.8)	110 (79.1)
Homework completion	26 (18.7)	63 (45.3)	48 (34.5)
Criminal justice data (inmate registries, WebCrims, etc)	25 (18.0)	59 (42.4)	55 (39.6)

### Expectations From Digital Technology in Psychiatry

**Figure 3:**
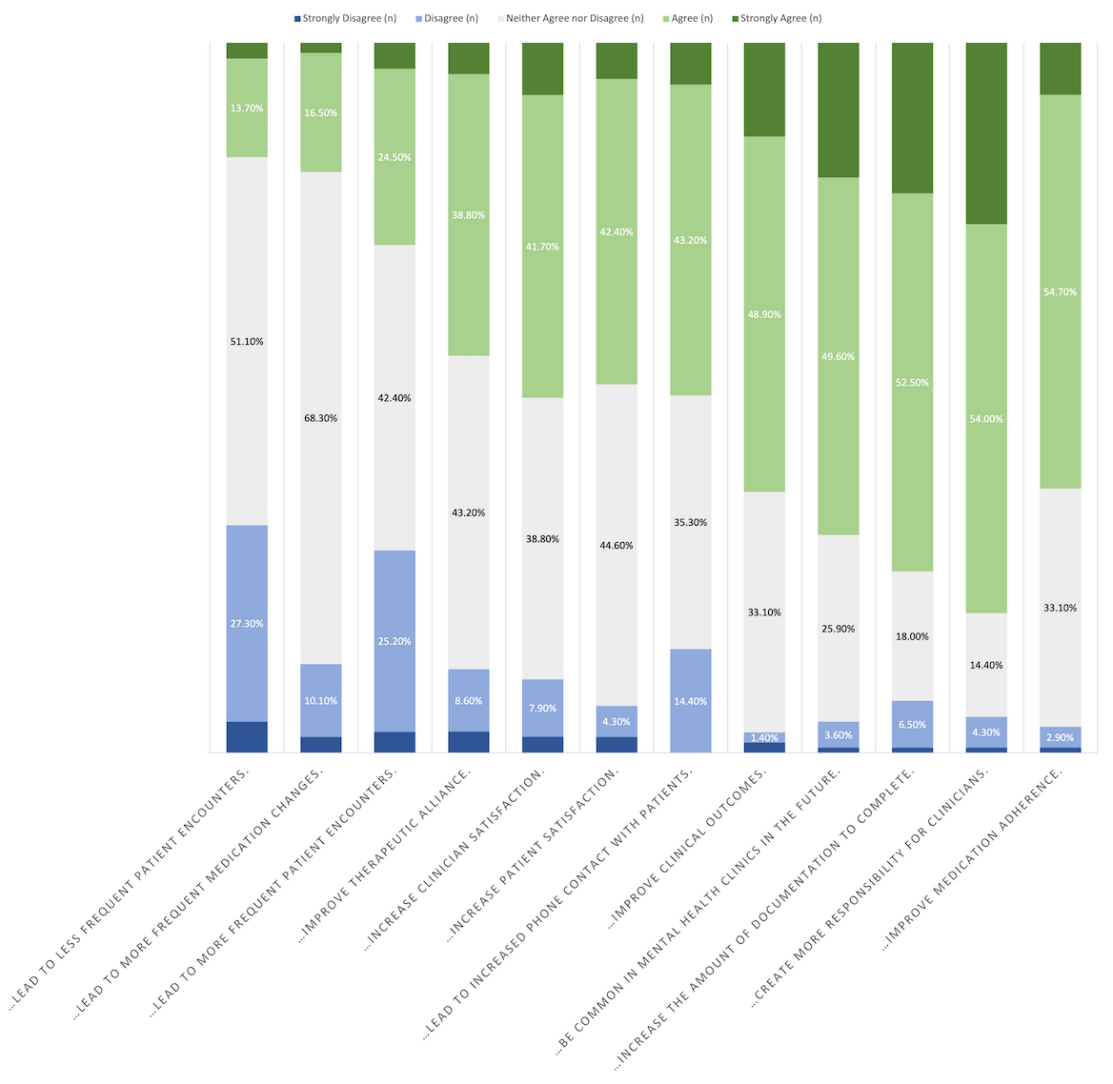
Clinicians' responses to the following statement: "Please rate your level of agreement with the following statements: Having access to information collected in a digital clinic will…".

Responses skewed negatively regarding whether or not the introduction of digital technology into psychiatry would change the frequency of patient encounters (“...lead to more frequent patient encounters”: 39, 28.1% respondents either disagreed or strongly disagreed; “...lead to less frequent patient encounters”: 44, 31.7% either disagreed or strongly disagreed). Responders expressed agreement that digital technology could “...Increase the amount of documentation to complete” and “...Create more responsibility for clinicians” (n=102, 73.4% and n=110, 79.2%, respectively; Figure 3).

Regarding past use of digital data in psychiatric practice, 32 (23.0%) participants responded affirmatively that they had viewed a patient’s social media accounts (Facebook, Twitter, Instagram, and other social media accounts) while delivering care. A total of 21 (33.9%) of those who answered yes cited gaining collateral information as the rationale, while others reported viewing social media to evaluate the clinical significance of the posts, to evaluate patients’ interpretations of posts, or for use in psychoeducation or therapeutic skill-building. In total, 120 (94.5%) responders stated that they thought their patients would be willing to share at least some digital data. Furthermore, 79 (63.2%) responders expressed agreement that digital technology in psychiatry could improve medication adherence.

Additional analyses were completed to assess differences in response patterns based on age and prescriber versus nonprescriber status (prescriber: MD physician, resident, or nurse practitioner; nonprescriber: nurse or social worker). There were overall few significant differences between groups, but younger responders expressed greater willingness to consult a digital dashboard (*P*=.01) and greater concern about increased time demands from digital data (*P*=.04) and expected greater patient satisfaction from a digital clinic (*P*=.04), and prescribers expressed greater interest in monitoring social media activity for posts concerning mental illness (*P*=.02) and greater uncertainty about how to respond to flagged digital events (*P=*.03). Younger responders were likelier to report having looked up a patient’s social media accounts (*P*=.005).

### Changes in Opinion After the COVID-19 Pandemic

When asked if the COVID-19 pandemic had affected their attitudes toward digital technology in psychiatry, 79 (56.8%) responders answered “yes.” Regarding how their attitudes had changed, responders provided a variety of responses in a free-text portion of the survey. Many were optimistic about digital technology in psychiatry after having had a positive initial experience with telepsychiatry: “More positive, more interest, greater comfort with telepsychiatry that makes me appreciate how digital data could inform my practice”; “It has normalized the use of technology in routine encounters”; “COVID is a disaster but I hope that a silver lining is that Telepsychiatry and digital psychiatry becomes more the accepted norm. Wider use of telepsychiatry is an excellent way to address the national shortage of psychiatrists available to deliver care”; and “Working from home greatly improves my work-life balance as a clinician. I find that patients feel the same way as they no longer have to travel to clinic, take time off work, or wait to see the doctor -- appointments start on time. For the stable, relatively high functioning outpatients that comprise my (very small) patient panel, telemedicine is equivalent to, and in many ways better than, in person psychiatry.” Some expressed skepticism, stating that “less interaction with the patient; not the same as in person visits.”

## Discussion

### Principal Findings

The purpose of our study was to examine clinician attitudes toward the use of digital data in mental health care via a survey of clinicians on the campus of an academic psychiatric hospital. We collected 139 survey responses. Overall, 83.4% (n=116) of responders reported positive expectations about digital data in clinical practice. Responders reported the highest enthusiasm for patient self-monitoring technologies and the strongest concern about potential increases in workload and actionability of digital data. As the use of digital data continues to gain prominence in mental health care, the results of our survey serve as a useful indicator of clinician expectations and concerns regarding this new technology in mental health. This study examined clinician attitudes toward the usefulness of different forms of digital data in clinical practice, clinicians’ anticipated benefits and barriers to employing digital technology in clinical practice, expectations of the ways in which clinical practice will change with the inclusion of digital data in psychiatry, experiences using patients’ social media data in practice, and the ways in which clinician perspectives on digital data in psychiatry have changed in response to the COVID-19 pandemic and the wide-scale shift to telehealth practice in medicine. Overall, the surveyed clinicians expressed enthusiasm to include digital data in their clinical practice and confidence that patients would be willing to share data; however, they expressed strong concern about increases in workload related to the inclusion of new technology in clinical practice.

### Comparison to Prior Work

While much attention has been paid to research developments, expert consensus, and patient expectations on digital data in psychiatry, clinician attitudes remain under-reported [8,9,11,20,21]. Our study offers a unique contribution to the literature on this rapidly developing area of mental health care, detailing clinician expectations of forthcoming digital technology in psychiatry, attitudes regarding how digital data can both help and hinder their practice, and current clinician behaviors regarding patients’ digital information (via web-based or social media search) at a unique point in time. These results will be useful in designing future digital clinics and be instructive for those disseminating digital data research as to which technologies clinicians are ready to embrace or remain reluctant.

Survey responders reported largely high expectations that digital data could improve their clinical practice, and more than half of them reported a shift in attitude after the onset of the COVID-19 pandemic, when most clinical practices had transitioned to a telehealth model. Providers also expressed high willingness to consult a digital dashboard and have patients do the same. These findings are consistent with the broader literature on the topic but higher than clinician expectation statistics reported elsewhere [20,22,23]. It may be that this enthusiasm reflected changing attitudes related to the COVID-19 pandemic or could be a reflection of the relatively young age of the group surveyed. Similarly, responders reported high confidence that patients would be willing to share digital data, consistent with other similar studies [23]. Our findings indicate that enthusiasm for the use of digital data in psychiatry and high expectations among clinicians that patients will feel the same way. Although there is a known gap between research enthusiasm and clinician enthusiasm [8,11], our data indicate that clinicians remain enthusiastic about digital data in psychiatry. This study should serve as encouragement for those seeking to implement digital technology into clinical practice.

Regarding the anticipated usefulness of different types of digital data in psychiatry, responders ranked items including sleep, substance use, patient-rated symptom scales, and physical activity as having high utility, but they rated location, internet search activity, and criminal data as having substantially less utility. One interpretation of this finding is that clinicians favor monitoring actionable metrics that could allow for discrete intervention over other types of digital measurement. This sentiment would appear echoed in the finding that clinicians rated “Helping patients track their activities and symptoms” and “As a support for clinical intervention” as the two most prominent benefits of using digital monitoring in mental health care. Previous work on digital technology in psychiatry has acknowledged a gap between what is notable in research versus what is readily adopted in clinical practice [8,11]. Our results indicate something similar and underscore the need to demonstrate clinical utility to encourage clinician adoption. Similarly, survey responders expressed concern about increases in documentation with the adoption of digital technology in clinical practice. In addition to demonstrating the actionability of digital data, designers of future clinical platforms will have to streamline the modes in which data are presented to clinicians to minimize the documentation burden.

The response to the question, “Have you ever viewed a patient’s social media (Facebook page, Twitter account, Instagram account etc)?” is a notable finding in itself because this behavior is extremely underreported in the literature [24,25]. Based on available research, our results are consistent with those of other studies [26]. Although not a strict equivalent to consulting a digital dashboard in the clinical setting (for both practical and ethical reasons), looking up patients on social media can be viewed as somewhat of a proxy for current use of digital data in clinical practice among those whom we surveyed. By that standard, despite their enthusiasm for new technology, our responders’ use of social media data was low. Given the controversy regarding this practice, it is plausible that our responders underreported how often they viewed patients’ social media accounts. Future research of other psychiatric clinicians in other settings could help to further elucidate how clinicians incorporate patient social media data into their assessments.

### Limitations

This study had several limitations. The first and most significant limitation is that it was conducted on a campus on which psychiatric digital technology research has had high visibility for several years. Although few responders identified themselves as researchers, many had likely had exposure to emerging digital technology and may have been predisposed to a positive response regarding potential benefits and lower trepidation about potential barriers of this technology in practice. This limits the generalizability of our data, when compared to clinician attitudes in community or nonacademic settings. Second, the survey was composed largely of closed-ended questions. Though there were opportunities for free-text responses where responders could express thoughts or feelings not captured in the survey questions, this mode of surveying may have left some attitudes unaccounted for.

A third limitation of our survey is timing, and the limitation is 2-fold. First, clinicians completed the survey beginning in May 2020, several months after a wide-scale shift to telehealth on our campus. In their free-text responses, many mentioned positive experiences with telepsychiatry but less specifically any of the digital technology described in the survey. It is possible that some responders conflated their experience with telepsychiatry with the technology described as part of the hypothetical digital clinic and provided contrived, more positive responses as a result. Future research must investigate how clinicians’ attitudes toward technology have continued to evolve in the postpandemic world. The second issue is that emerging technology is a dynamic, ever-shifting field, and that the generalizability of survey results, such as that of our survey, is limited to the moment in time the survey was administered. This limits the generalizability of our study or rather any study that discusses this topic. Lastly, the dimensions of our survey were not calculated, and specific validity and reliability tests were not conducted. Studies similar to this one will remain useful for understanding clinicians’ perspectives on these issues, but so will studies that measure the safety and efficacy of how these technologies are implemented in clinical practice.

### Conclusions

Our survey results indicate that clinicians’ attitudes toward the implementation of digital data in psychiatry are largely positive. However, responders voiced some trepidation about the actionability of digital data and increased time demands from addressing or documenting data. Overall, 23.0% (n=32) of responders reported having looked up patients on social media. More than half of the responders reported a change in attitude toward digital data in psychiatry following the COVID-19–related transition to telepsychiatry service models. Our survey results underscore the need for clinician engagement and education as digital data platforms are developed for clinical use in psychiatry.

## Appendix

Multimedia Appendix 1 Sample of the digital clinic clinician survey.
