# Supplementary material for: Digital Technology in Psychiatry: Survey Study of Clinicians
Source: JMIR Form Res. 2022 Nov 10;6(11):e33676. doi: 10.2196/33676 (PMC9693695; doi:10.2196/33676)
Supplement: Multimedia Appendix 1 [file formative_v6i11e33676_app1.pdf]

Background

Digital Psychiatry is a promising new field of mental health care and will soon be present on our campus via the [redacted] Digital Clinic. Mood-reporting and activity-tracking apps have increasingly been embraced by the public, and research is ongoing into how smartphone and social media activity can predict relapse/rehospitalization in psychiatric patients. The purpose of this survey is to measure your attitudes and expectations towards these technologies as they are integrated into clinical care.

Participation is voluntary. If you would prefer not to participate, simply return the blank survey. If you agree to participate, please complete the attached survey. Your responses are anonymous; do not put your name or other identifying information on this survey unless you would like to participate in a focused interview regarding the Digital Clinic. We ask that you try to answer all questions. However, if there are any questions that you would prefer to skip, simply leave the answer blank.

You must be at least 18 years old to participate. If you are not 18 or older, please inform the researcher and do not complete the survey.

This research has been reviewed by the Institutional Review Board (IRB). If you have any questions about your rights as a participant, or if you feel that your rights have been violated, please contact the IRB at [redacted].

Researcher Contact Information:
Dr. [redacted]
[redacted]

1. Could digital data about your patients' activities inform your treatment?

- ◯ No
- ◯ Yes
- ◯ I'm not sure, I need to know more
- ◯ Definitely, I incorporate this data already
- ◯ I think so, but would need to try it out

2. Please rate the relative levels of usefulness you think each category of information would have for your practice:

	Low	Medium	High
Sleep	◯	◯	◯
Physical activity	◯	◯	◯
Social media activity (frequency of posts or content of posts concerning for symptoms of mental illness)	◯	◯	◯
Online search activity (content of which is concerning for symptoms of mental illness)	◯	◯	◯
Mobility (time spent away from home versus at home)	◯	◯	◯
Location	◯	◯	◯
Substance use	◯	◯	◯
Self-reported symptom ratings	◯	◯	◯
Screen time	◯	◯	◯
Treatment adherence	◯	◯	◯
Homework completion	◯	◯	◯
Criminal justice data (inmate registries, ◯	◯	◯ Webcrims etc)	

3. Would you consult a dashboard of patient digital data prior to or during an appointment?

- Yes
- Maybe once in a while
- No

4. What do you anticipate would be the greatest barriers against your use of digital data?

	Not a barrier	Somewhat of a barrier	A significant barrier
Uncertainty about how to integrate digital data into practice			
Uncertainty about how to respond to flagged digital events			
Increased time demands tending to digital data during clinic visits			
Lack of trust in digital data			
Volume of data created by digital monitoring			
Increased documentation			
Invasion of patient privacy			
Training for patients			
Training for clinicians			
Increased time demands tending to flagged digital events			
Patient participation			
A lack of useful interventions for flagged digital events			
Interference with alliance			

Other (please specify)

5. What do you anticipate would be the greatest benefits of digital data?

	Not a benefit	Somewhat of a benefit	A significant benefit
Having a consistent source of collateral data	◯	◯	◯
Being able to monitor patient well-being between appointments	◯	◯	◯
Helping patients track their activities and symptoms	◯	◯	◯
Helping patients feel better understood	◯	◯	◯
As a support for clinical intervention (e.g., medication reminders, thought logs)	◯	◯	◯
As an alert system when patient activities change	◯	◯	◯
As a tool for initiating conversations with patients	◯	◯	◯

Other (please specify)

6. Have you ever viewed a patient’s social media (Facebook page, Twitter account, Instagram account etc)?

- Yes
- No

7. If you answered yes to Question 6, what have been your goals in looking at patient social media (please select all that apply)?

- [ ] For context about an anecdote the patient is relating
- [ ] For collateral information about a patient
- [ ] To evaluate the clinical significance of a post
- [ ] To evaluate the clinical significance of a patient's interpretation of a post
- [ ] N/A
- [ ] Other (please specify)

8. If you answer yes to Question 6, how did you make use of the social media you observed? (please select all that apply)

- [ ] As collateral data about the patient’s clinical status
- [ ] As the focus for a conversation with the patient
- [ ] As a tool for psychoeducation/skill building
- [ ] N/A
- [ ] Other (please specify)

9. Do you think your patients would share their digital data?

- Yes
- No
- Probably some types of data, but not all

10. Do you think it would be beneficial for patients to have access to an app-based dashboard with information about their digital data?

- ◯ Yes
- ◯ No

11. If you answered no to Question 10, why not?

12. Please rate your level of agreement with the following statements:

Having access to information collected in a Digital Clinic will…

	Strongly disagree	Disagree	Neither agree nor disagree	Agree	Strongly agree
…Lead to more frequent patient encounters.	◯	◯	◯	◯	◯
…Lead to less frequent patient encounters.	◯	◯	◯	◯	◯
…Lead to increased phone contact with patients.	◯	◯	◯	◯	◯
…Lead to more frequent medication changes.	◯	◯	◯	◯	◯
…Improve medication adherence.	◯	◯	◯	◯	◯
…Increase patient satisfaction.	◯	◯	◯	◯	◯
…Increase clinician satisfaction.	◯	◯	◯	◯	◯
…Improve clinical outcomes.	◯	◯	◯	◯	◯
…Increase the amount of documentation to complete.	◯	◯	◯	◯	◯
…Create more responsibility for clinicians.	◯	◯	◯	◯	◯
…Improve therapeutic alliance.	◯	◯	◯	◯	◯
…Be common in mental health clinics in the future.	◯	◯	◯	◯	◯

Demographics

Thank you for your participation - please tell us a bit about yourself!

13. Age?

[ ]

14. Gender?

- ◯ Male
- ◯ Female
- ◯ Decline to answer
- ◯ Other (please specify)

[ ]

15. Role in clinic?

- ◯ Psychiatrist
- ◯ Resident/Intern
- ◯ Psychologist
- ◯ Nurse Practitioner
- ◯ Nurse
- ◯ Social Worker
- ◯ Other (please specify)

[ ]

16. Department? (please check all that apply)

- ☐ AOPD
- ☐ Centers
- ☐ Inpatient
- ☐ ED
- ☐ PHP
- ☐ CAP
- ☐ Other (please specify)

[ ]

17. Years in practice? (Residents, use numbers 1-4 to indicate year of training)

[ ]

18. What type of care do you provide? (please check all that apply)

- [ ] Medication Management
- [ ] Individual Psychotherapy
- [ ] Group Therapy
- [ ] Neuromodulation
- [ ] Administration
- [ ] Other (please specify)

19. Which diagnoses do you primarily treat? (please check all that apply)

- [ ] Schizophrenia Spectrum and Other Psychotic Disorders
- [ ] Bipolar and Related Disorders
- [ ] Depressive Disorders
- [ ] Anxiety Disorders
- [ ] Personality Disorders
- [ ] Obsessive-Compulsive and Related Disorders
- [ ] Trauma- and Stressor-Related Disorders
- [ ] Disruptive, Impulse-Control and Conduct Disorders
- [ ] Substance-Related and Addictive Disorders
- [ ] Neurocognitive Disorders
- [ ] Neurodevelopmental Disorders
- [ ] Other (please specify)

20. Have your feelings about digital psychiatry changed in response to COVID?

- ( ) Yes
- ( ) No

21. If yes, how have your feelings changed?

22. Thank you for your participation in this survey, would you be interested in participating in a focused interview about the digital clinic?

- ◯ Yes
- ◯ No

23. If you answered yes to Question 20, please give us the best contact information by which to reach you:

**Name**	
**Email Address**	
**Phone Number**	
